# Delayed secretory activation and low milk production in women with gestational diabetes: a case series

**DOI:** 10.1186/s12884-022-04685-0

**Published:** 2022-04-22

**Authors:** Majed A. Suwaydi, Mary E. Wlodek, Ching Tat Lai, Stuart A. Prosser, Donna T. Geddes, Sharon L. Perrella

**Affiliations:** 1 School of Molecular Sciences, The University of Western Australia, Crawley, WA Australia; 2 Department of Medical Laboratory Technology, College of Applied Medical Sciences, Jazan University, Jazan, Saudi Arabia; 3 One For Women, Mt Lawley, WA Australia

**Keywords:** Gestational diabetes mellitus, Breastfeeding, Lactation, Human milk, Case series

## Abstract

**Background:**

Gestational diabetes mellitus (GDM) is major pregnancy complication that is associated with short- and long-term consequences for both mother and infant, including increased risk of diabetes later in life. A longer breastfeeding duration has been associated with a reduced risk of diabetes, however, women with GDM are less likely to exclusively breastfeed and have shorter breastfeeding duration. While the timing of breastfeeding initiation and milk removal frequency affects subsequent breastfeeding outcomes, little is known about early infant feeding practices and milk production in women with GDM. This case series offers detailed prospective breastfeeding initiation data, as well as the first report of objective measures of milk production in women with GDM.

**Case presentation:**

In this case series, we present the early infant feeding practices of eight women with GDM that gave birth at term gestation. Women recorded the timing of initiation of breastfeeding and secretory activation, as well as their breastfeeding, expression and formula feeding frequencies on postpartum days 1, 7 and 21. Measurement of 24 h milk production volume was performed at 3 weeks postpartum using the test weight method. We observed a delayed first breastfeed (> 1 h) in 6 (75%) cases, formula use in hospital in 5 (63%) cases and delayed secretory activation in 3 (38%) cases. At 3 weeks postpartum, 2 cases had measured milk productions that were insufficient to sustain adequate infant weight gain.

**Conclusions:**

Our data suggest that despite early and frequent milk removal, women with GDM are at greater risk of delayed secretory activation and low milk supply. Cohort studies that consider co-morbidities such as obesity are needed to determine the lactation outcomes of women with GDM.

## Background

Gestational diabetes mellitus (GDM) is defined as impaired glucose tolerance with the first onset during pregnancy [1]. Although GDM commonly resolves after pregnancy, exposure to GDM is associated with short- and long-term consequences for mothers and their infants, including maternal risk of developing type 2 diabetes (T2D) 10 times higher than women without GDM [2]. Furthermore, GDM may impact the lifetime risk of offspring, fuelling a vicious circle of offspring obesity, impaired glucose metabolism, and metabolic syndrome that contribute to the subsequent development of T2D later in life [3, 4].

The postnatal period is an important window for metabolic programming in setting future chronic disease risk [5]. There is evidence that continued breastfeeding after a pregnancy complicated by GDM reduces the risk of developing T2D for women and their infants [6, 7]. However, breastfeeding difficulties are common among women with GDM, with lower rates of predominant breastfeeding on hospital discharge [8, 9] and shorter breastfeeding duration [10, 11]. Indeed, results from a recent systematic review and meta-analysis indicate that GDM-exposed infants had 40% higher rates of formula supplementation in hospital, 1 month shorter duration of breastfeeding, and a 30% decrease in breastfeeding rates at 12 months [12].

Milk production is dependent on appropriate breast development in pregnancy. Insulin metabolism has been reported as an important regulator of milk secretion, playing a major role in the mammary gland switch from proliferation to differentiation. Insulin upregulates genes associated with mammary epithelial cell (MEC) proliferation and downregulates genes related to MEC differentiation [13, 14]. Therefore, lower insulin sensitivity in diabetes may be associated with impaired secretory differentiation, with subsequent delayed secretory activation and reduced milk production [15].

Early infant feeding practices impact subsequent milk production [16], and so may confound the breastfeeding outcomes of women with GDM. Evidence from healthy mothers with term infants suggests that on-demand breastfeeding ≥8 times per 24 h is positively associated with milk production; therefore, reduced milk removal frequency during the early postpartum days and weeks may compromise the 24 h milk production [16, 17]. The existing evidence regarding early infant feeding practices and milk production is limited in this population. Therefore, this case-series aimed to examine early infant feeding practices among women with GDM, measure 24 h milk production and report breastfeeding outcomes at 3 weeks postpartum.

## Case presentation

Women with GDM were recruited from a maternity care clinic “One For Women” in Perth, Western Australia, and provided informed consent to participate in an observational study (UWA Human Research Ethics, RA/4/20/5657). Women were diagnosed with GDM based on the Australasian Diabetes in Pregnancy Society criteria using the oral glucose tolerance test (OGTT) between 24 and 28 weeks of gestation [18]. Maternal demographic data were collected at enrolment, including age, ethnicity, education, medication use, parity, and smoking status. Bra cup sizes before and during pregnancy were recorded to detect breast growth. Height and pre-pregnancy weight were collected to determine pre-pregnancy body mass index (BMI).

Lactation related data were also collected including breast surgery, previous and intended breastfeeding durations. Birth mode, birth gestation, infant anthropometrics and sex were recorded. Detailed breastfeeding data were collected at 1, 7 and 21 days postpartum. Early initiation of breastfeeding was defined as putting the newborn to the breast within 1 h of birth, irrespective of infant suckling or milk transfer from the breast infant [19]. The timing of the first breastfeed (infant attached and suckled at breast) and timing of first breast expression (by hand or pump), as well as frequency of milk removal in the first 24 h were recorded. We combined these data to report timing of first milk removal, and frequency of milk removal was reported as the sum of breastfeeding and expression sessions over a 24 h period. Secretory activation is the onset of copious milk secretion that typically occurs at 48 – 72 h after birth and is reported clinically as the time of milk ‘coming in.’ Different milk biomarkers indicate the onset of secretory activation between 7 and 24 h before the mother’s perception of her milk “coming in” or breasts being fuller [20, 21]. However we used maternal perception of milk coming in as published reviews have concluded this is valid and feasible indicator of the onset of secretory activation [21–23]. Onset of milk “coming in” after day 3 postpartum was considered to indicate delayed secretory activation.

Maternal milk production was measured at 3 weeks postpartum using the 24 h test weigh method. Mothers use an electronic scale (BabyWeigh; Medela, McHenry, IL; resolution, 2 g; accuracy, 0.034%) to measure their infants’ weight before and after each feed and the volume of any milk expressed over a 24 h period [24]. Emerging evidence suggests that a full milk supply may be achieved at 2 weeks postpartum [25]. The mean 24 h milk production of fully breastfeeding dyads at 1 – 6 months postpartum is 788 ± 169 mL/24 h, with low milk production classified as < 600 mL/24 h [24]}.We did not use a stable isotope technique such as deuterium dilution, because it requires repeated sampling of maternal and infant saliva over 14 days, and (expensive) analysis of isotopes [26]. Further, stable isotope technique measurements correlate with 24 h test weights when electronic scales are used [27]. The 24 h test weight method offers the benefit of additional data including frequency of milk removal by breastfeeding and/or pumping, and volumes of any supplementary feeds given.

### Outcomes

#### Maternal and infant characteristics

Eight women participated in a study designed to examine infant feeding practices and 24 h milk production after pregnancies complicated by GDM. Maternal and infant characteristics are reported in Table 1. All eight women had intended breastfeeding durations of at least 12 months and the multiparous case had previously breastfed for 12 months. Three cases were within the healthy BMI range, one was overweight, and four were obese. Histories of anxiety (*n* = 4) and depression (*n* = 2) were reported. None had previous breast surgeries or smoking history. Three had medical histories of polycystic ovary syndrome (cases D and E) and endometriosis (case E). Pregnancy complications other than GDM were shortened cervix (case F), and anemia (case G). All gave birth at term gestation, and one had a postpartum haemorrhage. No other birth complications were reported. The median infant birth weight was 3310 g (range 2590 - 4000 g), and four (50%) infants were male.

**Table 1:** Maternal and early feeding characteristics

Characteristics	Case A	Case B	Case C	Case D	Case E	Case F	Case G	Case H
Maternal age (years)	39	34	30	36	31	37	30	35
Parity	3	1	1	1	1	1	1	1
Pre-pregnancy BMI (kg/m2)	24	28.6	36.2	42.7	33.3	34.9	24.8	24
Pregnancy OGTT result (mmol/l)								
Fasting	4.5	4.1	4.3	5.6	4.5	5.3	4.6	4.3
1 h	10.5	10.3	8	11.2	8.7	11.8	9.1	9
2 h	10.8	6.2	8.9	8.3	8.6	6	8.5	9.7
Bra cup size change in pregnancy	+ 1	+ 1	−1	+ 2	+ 1	+ 1	+ 2	+ 1
Birth gestational age (weeks)	38.7	38.1	39.4	38	38.3	37.6	39.7	40.1
Birth mode (vaginal = V, Caesarean section = CS)	V	V	V	CS	V	CS	V	CS
Neonatal nursery admission	No	Yes^a^	No	No	No	No	No	No
Timing of first breastfeed (h)	1.5	0.16	1	> 2	NA^b^	> 2	1.16	1.5
Timing of first breast expression (h)	2	5	5	2	2	3	5	NA^c^
Infant fed formula in hospital	Yes	Yes	No	Yes	Yes	Yes	No	No
Timing of milk coming in (days)	8	4	5	5	3	3	3	4

*BMI* body mass index, *OGTT* oral glucose tolerance test

^a^Infant admitted to neonatal nursery at 11 h of age for management of hypoglycaemia (blood glucose level 1.9 mmol/L) and discharged to mother after 12 h

^b^Infant was unable to attach to the breast during the postnatal hospital stay

^c^Breast expression was not performed during the postnatal hospital stay

Early initiation of breastfeeding and timing of first breastfeed: All infants had contact with the breast within an hour of birth and so all achieved early initiation of breastfeeding according to the World Health Organization definition [19]. However, for 6 cases the first breastfeed was delayed > 1 h after birth: cases A, G, and H first breastfed at 1.5, 1.16, and 1.5 h after birth, respectively. Cases D and F first breastfed > 24 h postpartum, and case E did not breastfeed during the hospital stay due to latching difficulties. Seven women commenced breast expression within 2 to 5 h of birth. The interval between birth and first removal of milk from the breast (by breastfeeding or expression) ranged from 0.16 to 3 h (Table 1).

Milk removal frequency: Frequencies of milk removal on days 1, 7, and 21 postpartum were median (range) 9.5 (3 - 17), 8.5 (7 - 11), and 8 (7 - 12), respectively (Table 2).

**Table 2:** Feeding practices and 24 h milk production in women with gestational diabetes mellitus

	Day Postpartum												
	1	7	21	1	7	21	1	7	21	1	7	21	21
	Freq MR^**a**^			BF^**b**^			Expression^**c**^			Formula use			24 h MP^**d**^
Case A	15	9	7	8	3	0	7	6	7	Y	N	Y	834
Case B	17	9	11	10	8	9	7	1	2	Y	N	N	903
Case C	11	8	7	6	0	8	5	8	7	Y	Y	Y	215
Case D	3	8	12	0	0	13	3	8	5	N	N	N	776
Case E	8	8	8	0	0	7	8	8	8	Y	N	N	1210
Case F	6	7	9	2	2	8	4	5	4	Y	Y	Y	326
Case G	9	11	7	7	11	7	2	0	0	N	N	N	613
Case H	10	9	8	10	8	8	0	1	0	N	N	N	636

*Y* yes infant fed formula, *N* no infant not fed formula

^a^frequency of milk removal from the breast (sum of breastfeeding and expression frequencies)

^b^breastfeeding frequency

^c^breast expression frequency using hand or pump

^d^24-hour milk production (mL)

#### Secretory activation

The reported timing of milk ‘coming in’ ranged from 3 to 8 days postpartum, with delayed secretory activation reported in 5 cases (Table 1). Of those, 3 were overweight or obese, and 6 achieved a 24 h milk production within the reference range by week 3 (Table 2).

#### Milk production

At 3 weeks postpartum, 2 women had low 24 h milk production (< 600 mL/24 h); both were obese (Table 2). Another two exclusively breastfeeding women had milk productions close to 600 mL/24 h; their infants’ WHO weight-for-age centiles at birth and 3 weeks were 88th and 55th centile, and 94th and 75th centile respectively. The case with the longest delay in secretory activation (8 days postpartum) had a milk production of 834 mL/24 h and was using a galactagogue and formula supplementation. There were no obvious trends between OGTT results and milk production, however the small sample size precluded statistical analysis.

#### Exclusivity of breastfeeding

Exclusive breastfeeding was reported for 3 cases and mixed feeding from birth was reported for 3 cases. Five of the 8 cases reported infant formula supplementation in hospital (Table 2). Of the 2 cases that were feeding infant formula on days 7 and 21 postpartum, one had commenced formula use in hospital, and both had low milk production confirmed by measurement.

## Discussion

This case series reported the early breastfeeding patterns and 24 h milk production of eight women following pregnancies complicated by GDM. Our observations showed a delay in timing of the first breastfeed, delayed secretory activation, and low milk production within a sample of women who had GDM, despite strong breastfeeding intentions and frequent milk removal at postpartum days 1, 7 and 21.

In this case series, commencement of breastfeeding > 1 h after birth was observed in 6 of 8 cases. Breastfeeding within an hour of birth is considered very important to the establishment of breastfeeding in healthy dyads with uncomplicated pregnancies [28]. Limited and conflicting evidence is available on the timing of the first breastfeed in women with GDM; one Vietnamese study reported no difference between women with and without GDM [10], while a North American study found significantly fewer mothers with GDM initiated breastfeeding within an hour of birth [29]. Women with GDM are likely at higher risk of delayed commencement of breastfeeding due to several inter-related factors, and so may need additional support to breastfeed within an hour of birth or to express colostrum when breastfeeding is not possible.

We observed 5 cases with delayed secretory activation despite 4 of these cases achieving early and frequent milk removal in the first 24 h after birth. Milk coming in after 72 h postpartum, or delayed secretory activation, is associated with GDM as well as other risk factors that may co-exist in women with GDM including pre-pregnancy overweight and obesity, caesarean birth, and medical management of neonatal hypoglycaemia such as early formula supplementation [30, 31]. These factors may contribute to reduced frequency of milk removal from the breast that is assumed to delay secretory activation, however the timing of secretory activation is not different in healthy women that choose not to initiate breastfeeding [32, 33]. It is possible that insulin resistance might be a potential mechanism that links GDM and delayed secretory activation. An animal model with modified MEC insulin receptors showed diminished mammary ductal growth and alveolar development [13]. Similarly, a recently published study used human-derived MECs for mammosphere culture to show the regulatory role of insulin in human MEC differentiation. The human mammosphere model suggests that insulin is essential for mammosphere formation by upregulation of differentiation, cell-cell junctions, and cytoskeleton organisation functions through integrin-linked kinase signalling [14]. The impact of biochemical aberrations on early mammary gland secretion is likely the causative mechanism of delayed secretory activation [33], with altered concentrations of biochemical markers of milk “coming in” such as lactose and citrate observed in women with diabetes [30]. However, the exact pathways that explain these outcomes are complicated and required further investigation.

Milk removal (breastfeeding) was delayed in most cases, however the case with the lowest milk production started breastfeeding within an hour of birth and had subsequent frequent milk removal. While lower milk production at 1 and 6 weeks postpartum has been linked to delayed and infrequent milk removal [16, 34], milk production also depends on intrinsic factors such as adequate functional glandular tissue and endocrine factors that impact proliferation and differentiation [16, 35]. Therefore, pathways contributing to the unintended breastfeeding outcomes of women with GDM are complex and challenging to interpret.

Despite regular milk removal, low milk production was measured in two women whose infants were supplemented with formula to maintain optimal weight gain (Table 2). Maintenance of milk removal ≥8 times per 24 h, whether by breastfeeding and/or pumping, typically enables the achievement of adequate milk production [33, 36]. Several factors may impact milk production, such as inadequate breast development, and possible biochemical aberrations associated with GDM and obesity. While it has been thought that social factors impact lactation in women with obesity, recent evidence supports endocrine factors, with the influences of progesterone and oestrogen within the mammary fat pad yet to be examined [37]. Large cohort studies are required to determine possible biochemical pathways for reduced milk production in women with obesity and GDM, while accounting for other lactation risk factors.

Our case series is limited by the small sample size that prevented controlling for co-existing lactation risk factors and measurement of an effect size. There is growing evidence for endocrine effects of both GDM and obesity on lactation. While it is not possible to differentiate the effects in a case series, observations from our study highlight the complexity of lactation in women with co-existing risk factors.

## Conclusion

Our observations of delayed secretory activation in 5 of 8 cases, and low milk supply in 2 of 8 cases emphasise the need for cohort studies to better understand the lactation challenges faced by women with GDM, including the examination of endocrine and molecular pathways that lead to delayed secretory activation and low milk production.

## Data Availability

The datasets generated and/or analysed during the current study are not publicly available to further preserve the confidentiality of the participants.

## Abbreviations

GDM: Gestational diabetes mellitus
T2D: Type two diabetes
MEC: Mammary epithelial cell
OGTT: Oral glucose tolerance test
BMI: Body mass index
PCOS: Polycystic ovary syndrome
